# The microRNA Cargo of Human Vaginal Extracellular Vesicles Differentiates Parasitic and Pathobiont Infections from Colonization by Homeostatic Bacteria

**DOI:** 10.3390/microorganisms11030551

**Published:** 2023-02-21

**Authors:** Paula Fernandes Tavares Cezar-de-Mello, Stanthia Ryan, Raina N. Fichorova

**Affiliations:** Laboratory of Genital Tract Biology, Department of Obstetrics, Gynecology and Reproductive Biology, Brigham and Women’s Hospital, Harvard Medical School, Boston, MA 02115, USA

**Keywords:** miRNA, *Trichomonas vaginalis*, *Prevotella bivia*, *Lactobacillus crispatus*, bacterial vaginosis, dysbiosis, exosome, female reproductive tract, estrogen receptor 1 (ESR1), glucocorticoid receptor (NR3C1)

## Abstract

The disturbed vaginal microbiome defined as bacterial vaginosis (BV) and the parasitic infection by *Trichomonas vaginalis* (TV), the most common non-viral sexually transmitted pathogen, have well-established adverse effects on reproductive outcomes and susceptibility to infection and cancer. Molecular mechanisms underlying these associations and the failure of antibiotic therapy to mitigate adverse consequences are not fully elucidated. In an *in vitro* human vaginal colonization model, we tested the hypothesis that responses to TV and/or BV-bacteria will disrupt the micro(mi)RNA cargo of extracellular vesicles (EV) with the potential to modify pathways associated with reproductive function, cancer, and infection. miRNAs were quantified by HTG EdgeSeq. MiRNA differential expression (DE) was established in response to TV, the BV signature pathobiont *Prevotella bivia* and a homeostatic *Lactobacillus crispatus* with adjusted *p* < 0.05 using R. Validated gene targets, pathways, protein-protein interaction networks, and hub genes were identified by miRWalk, STRING, Cytoscape, and CytoHubba. In contrast to *L. crispatus*, TV and the BV pathobiont dysregulated a massive number of EV-miRNAs, over 50% shared by both pathogens. Corresponding target pathways, protein interaction clusters and top hub genes were related to cancer, infectious disease, circadian rhythm, steroid hormone signaling, pregnancy, and reproductive tissue terms. These data support the emerging concept that bacteria and parasitic eukaryotes disturbing the human vaginal microbiome may impact reproductive health through EV-miRNA dysregulation.

## 1. Introduction

The influence of the human microbiome on health or disease is an evidence-based scientific consensus [1,2,3,4]. The intricate relationship between human host cells and a diverse multispecies community has been shaped throughout evolution and is key to understanding how a dysbiotic environment challenges human health. The lower and upper portions of the human female reproductive tract (FRT) are differentially colonized [5], while the lower FRT (including the vagina and uterine cervix) is more diversely populated by commensal and regular residents [6]. The vaginal microbiota has been classified by microbiome sequencing into five distinct community state types (CST) based on the predominance of certain lactobacillus species or bacterial pathobionts [7]. Conceptually, the ‘core microbiome’ [8] of a healthy vagina is featured by the predominance of three species of the genus *Lactobacillus* (*L. crispatus*—CSTI, *L. gasseri*—CSTII and *L. jensenii*—CSTV) [9] and by their ecological relationships with additional biotic components, plus intrinsic and exogenous factors. *L. crispatus*, *L. gasseri,* and *L. jensenii* can deliver a mucosal environment protective against ascendance of pathogens to the upper FRT [10]. Disturbances in this ecosystem may lead to a dysbiotic environment (CST-IV, 7–10 Nugent score and pH > 5), such as bacterial vaginosis (BV) syndrome, characterized by the reduction in lactobacillus species and majority of obligate anaerobic polymicrobial spectrum [6] where *Prevotella bivia* is the most common BV-associated pathobiont bacteria [11,12] prevailing along with *Gardnerella vaginalis*, and *Atopobium vaginae* [7]. Sequencing of the cervical microbiome also ranked the *L. crispatus*-dominated CST as the least proinflammatory, and the *P. bivia*-dominated as the most proinflammatory, hence, pathogenic CST [13]. BV is linked with an increased risk of developing urogenital cancer, obstetric complications, and acquiring sexually transmitted infections (STIs) [6], including human papillomavirus (HPV) [14,15], human immunodeficiency virus (HIV) [16,17] and *Trichomonas vaginalis* (*T. vaginalis*) [18], the causative agent of trichomoniasis.

Trichomoniasis is the leading global cause of non-viral STIs [19]. *T. vaginalis* may cause subclinical to asymptomatic infections in roughly 50% of women and over 70% of men, which contributes to underestimating global rates of infection and collaborates with a sustained transmission chain [20]. Like BV, *T. vaginalis*-infected individuals are at higher risk of acquiring other STIs, including HIV, and developing cervical and prostate cancer [20]. *T. vaginalis* infections also share with BV adverse effects on obstetrical outcomes, e.g., preterm birth (PTB), low birth weight, and premature rupture of membranes [20,21,22]. Although curable, *T. vaginalis* and BV-associated poor pregnancy outcomes may persist after antibiotic treatment and pathogen elimination [20,23]. The causation of the persistence of perinatal adversities after parasite clearance is uncertain. Multiple mechanisms of treatment failure have been suggested including innate immunity dysregulation due to endosymbiotic *T. vaginalis* virus (TVV) shed by the parasite during antibiotic treatment [24] and modification of the vaginal microbiome by the parasite which may result in persistent BV [6,25,26]. We hypothesize that a homeostatic failure following *T. vaginalis* infection could be at least in part driven by the host epigenome dysregulated by both the parasite and BV pathobionts. 

A key element of the epigenome regulated by host-microbe interactions is represented by the human micro(mi)RNA transcriptome (miRNome). miRNAs are small single-strand (~22nt long) non-coding RNAs known to negatively modulate gene expression at the post-transcriptional level. The canonical way of miRNA-led post-transcriptional regulation is by promoting mRNA degradation or translational repression [27] by base paring the 5′-proximal seed region (nucleotide 2 to 8) of the miRNA with mRNA 3′UTR [28,29], ultimately leading to gene silencing [29]. miRNA expression is sensitive to myriad pathologies; therefore, investigating such dysregulation would provide opportunities to understand the molecular mechanism underlying health and disease, identify miRNA:mRNA therapeutic targets of high importance, and relevant biological process. Although miRNA expression may be cell/tissue-specific, they eventually will be exchanged between cells to exert paracrine regulation. MiRNAs also stably circulate in many body fluids in the form of protein complexes or encased in extracellular vesicles (EV)—groups of right-side-out membranous vesicles which differ in size and cellular biogenesis origin. Due to its specific and selected cargo—a cocktail of proteins, lipids, nucleic acid, and glycans—EVs are mediators of same-species and cross-kingdom cell-cell communications [30], and hence, lend themselves as players of post-transcriptional and potentially epigenetic regulation directed by EV-contained miRNAs (EV-miRNAs). A growing number of studies have aimed to characterize the interplay of EVs in relevance to parasite-host interactions [31], including *T. vaginalis* [32,33,34,35]. However, there is a lack of studies on EV-miRNAs released by the human vaginal epithelium in response to parasitic infection and no comparative causative evaluation of vaginal pathogens and homeostatic bacteria. 

In this study we tested experimentally the hypothesis that colonization of human vaginal cells by *T. vaginalis* and/or BV-associated bacteria will disrupt the EV-miRNAs cargo, with the potential to target molecular pathways underlying some of the long-lasting systemic effects and reproductive disorders. Using a well-validated human *in vitro* infection model, we identified: (1) EV-miRNAs differentially released by vaginal epithelial cells upon disruptive (*T. vaginalis* or *P. bivia*) versus homeostatic (*L. crispatus*) colonization suggesting causality; and through *in silico* miRNA target prediction (2) genes and pathways of failed homeostasis along with disrupted protein-protein interaction networks with highest connectivity and hence highest potential to discover novel approaches to treatment and prevention of disease.

## 2. Materials and Methods

### 2.1. Experimental Model

Human vaginal epithelial cells (Vk2/E6E7) derived from a healthy tissue following vaginal repair surgery [36] were grown (6 × 10^5^ cells/mL) to 80–90% confluency in 6-well plates in antibiotic-free keratinocyte serum-free medium (KSFM) as described [37] and exposed to antibiotic-free KSFM control or monocultures of *T. vaginalis* (8 × 10^5^ CFU/mL), *L. crispatus* (7 × 10^6^ CFU/mL) and *P. bivia* (7 × 10^6^ CFU/mL) at 35 °C in Mitsubishi AnaeroPack chambers (Fisher) mimicking the human vaginal microenvironment [37]. The *T. vaginalis* clinical isolate (UR1) was chosen for these experiments for its well-characterized pathogenicity, including lipophosphoglycan composition [38,39,40,41], immune properties [24,35,38,39,42,43,44,45,46], and carriage of endosymbiont *Trichomonasvirus* species [24,42,44,46], thereby representing a common clinical infection. *L. crispatus* (#223-2-10) and *P. bivia* (#1–17) were previously isolated from human vaginal swabs [47] and confirmed to have homeostatic or respectively, disruptive immune impact on the vaginal epithelium [37,42,48,49].

After 24 h incubation, cell culture supernatants were collected for EV isolation and cells were harvested for cell viability and colonization assessment. Reproducible bacterial colonization was ascertained by epithelial-cell attached colony-forming units as described [37]. Trichomonas viability and motility were confirmed by microscopic observation from total counts obtained from supernatant pellets, epithelial monolayer washings and after epithelial monolayer trypsinization [24]. Epithelial cell viability was confirmed by microscopic evaluation as well as by Trypan blue exclusion test using an automated cell counter and repeated measures of percent viability for each condition were compared by ordinary one-way ANOVA, and Tukey’s multiple comparison test (GraphPad Prism 9.4.1 for Windows, GraphPad Software, San Diego, CA, USA). EVs pellets were collected from biological quadruplicates of each of the 4 exposures (medium, *T. vaginalis*, *L. crispatus* and *P. bivia*) in 3 independent experiments resulting in 48 biological replicates individually tested in 3 miRNA assay runs. In a fourth independent experiment, each exposure was repeated in 12 individual epithelial cell cultures and a single pool of 12 was generated for each exposure/experimental condition. Each of these pools was split into 6 aliquots (two of each tested in each independent miRNA run) resulting in 24 technical replicates for assessing technical reproducibility of the miRNA expression profiling between runs.

### 2.2. Small Extracellular Vesicles Isolation and Characterization

Cell culture supernatants were centrifuged at 1000× *g* for 5 min at 4 °C and filtered through a 0.22 µm membrane. Filtered supernatants were mixed in a 2:1 *v*/*v* ratio with the Total Exosome Isolation Reagent for cell culture medium (Invitrogen, Carlsbad, CA, USA) and incubated for 16 h at 4 °C followed by centrifugation using a fixed angle rotor at 10,000× *g* for 60 min at 4 °C. ZetaView (Particle Metrix, Meerbusch, Germany) was used to assess size and concentration of EVs and transmission electron microscopy (TEM) with immunogold labelling of CD63 was used to confirm the presence of exosomes in the EVs samples as previously described [35] (Supplementary Figure S1A). For miRNAs expression profiling, supernatants were removed, EV pellets were resuspended in 35 µL of HTG lysis buffer, and frozen at −80 °C until analyzed for the global human miRNA transcriptome.

### 2.3. Whole Human miRNA Transcriptome Profiling

The miRNA content in the experimental EV preparations was profiled using the high-fidelity HTG EdgeSeq platform (HTG Molecular Diagnostics Inc, Tucson, AZ, USA) which couples an RNA extraction-free nuclease protection assay with next generation sequencing to quantify the whole human miRNA transcriptome (miRNome). The platform has been rigorously evaluated in multiple disease conditions and by comparison to other sequencing methods [50,51,52,53,54,55]. HTG EdgeSeq Whole Transcriptome (WT) miRNA assay used for this study contained 2102 probes, including 13 housekeeper genes, 5 negative controls genes from *Arabidopsis thaliana aintegumenta* (ATN), and 6 positive process control. Quality control (QC) was performed based on the background values of the ANT genes. ANT were averaged for each sample and a grand mean was calculated by taking the average of these averaged ANT values. The difference between each averaged ANT value and the grand mean, Δmean (ΔMean = averaged sample mean—grand mean) and standard deviation (SD) of the Δmean were calculated for each sample. The acceptable Δmean values are those within ±2XSD average ANT. The majority of samples (71 of 72) passed the QC test and were used to generate miRNA data. For WT miRNA assay, 25 µL of each lysate was run on an HTG EdgeSeq Processor in 3 individual plates (blocks) of 24 samples, using the HTG EdgeSeq miRNA WT assay. Samples were randomized within each plate to avoid any potential location bias. Each plate contained a technical duplicate of each pool for each of the four exposures and a randomized set of biological quadruplicates. Following the processor step, samples were individually barcoded using a 16-cycle PCR reaction to add adapters and molecular barcodes. Barcoded samples were individually purified using AMPure XP beads and quantitated using a KAPA Library Quantification kit to generate a library pool. The library was sequenced on an Illumina MiSeq using a V3 150-cycle kit with two index reads. PhiX was spiked into the library at 5% as a standard procedure for Illumina sequencing libraries. Data were returned from the sequencer in the form of demultiplexed FASTQ files. The HTG EdgeSeq Parser was used to align the FASTQ files to the probe list to collate the data.

### 2.4. miRNA Transcriptome Bioinformatics and Statistical Analysis

Bioinformatics and statistical analyses were performed using R (V3.3.0). Normalization was conducted in DESeq2 (version 1.14.1) using the regularized log approach. The differential expression analysis of the EdgeSeq miRNA data was completed using the DESeq2 package (version 1.8.1) available from Bioconductor [56]. The DESeq2 package provides methods for estimating and testing differential expression using negative binomial generalized linear models. Empirical Bayes methods were used to estimate dispersion and log2 fold change (FC) with data-driven prior distributions. The DESeq2 model corrected for library size using the median ratio method [57]. Dispersions were estimated with the Cox Reid-adjusted profile likelihood method [58], and Log2 FC was estimated via Tikhonov/ridge regularization with a zero-centered normal prior distribution with variance, calculated using the observed distribution of maximum likelihood coefficients. DESeq2 performs independent filtering on probes prior to testing and application of the false discovery rate *p*-value adjustment to increase power.

Differential expression analyses were conducted using multiple regression accounting only for treatment, such that *p*-values were calculated comparing a full model (all treatment groups) to a reduced model containing only the intercept for each probe individually using a hierarchical likelihood ratio test (hLRT). To account for multiple tests, *p*-values from the hLRT were adjusted using the Benjamini & Yekutieli approach at an adjusted significance level of 0.05. Results were plotted as the log2 FC of the largest absolute value (x-axis) against the adjusted log10 *p*-value (y-axis) by probe. For the volcano pots, probes included were filtered by adjusted *p* < 0.05 and log FC > 2.

Multidimensional scaling (MDS) was used to evaluate similarities and differences in the EV-miRNome of non-colonized (control) versus colonized vaginal cells and the batch effect. This assessment was performed using the R *prcomp* package, without centering or scaling using the regularized log normalization, to create principal components explaining variance. Expression heatmaps were calculated based on the regularized log normalized data. Pairwise correlations were calculated using the Kendall tau coefficient and correlation heat maps were generated to visualize the pair-wise comparisons of technical replicates to demonstrate reproducibility over all run blocks/batches.

### 2.5. miRNA-Target Genes Prediction and Gene Set Enrichment Analysis 

Differentially expressed (DE) miRNAs (adjusted *p*-value < 0.05) were selected to generate lists of shared and non-shared EV-miRNAs by Venn diagram analysis [59], miRNA-target genes prediction and pathway-enrichment analysis. Putative miRNA-target gene pairs were predicted using miRWalk database (release_2022_01) [60]. Data returned validated miRNA-target genes trough stringent prediction criteria using three combined algorithms (TargetScan, miRDB, and miRTarBase). The target gene list was uploaded into STRING (V11.5) [61] to construct protein-protein interaction (PPI) networks and pathway-enrichment analysis using Kyoto Encyclopedia of Genes and Genomes (KEGG) and TISSUES databases. To this end, we applied the data settings highest confidence interaction score > 0.9 and limited the number of interactions to the queried proteins; except for groups with a small number of predicted targets (17 predicted targets for EV-miRNAs down-regulated by both *T. vaginalis* and *P. bivia*, and 3 predicted targets for EV-miRNAs up-regulated by *P. bivia*) where we used number of interactions = no more than 5. PPI network clusters were built using Markov Clustering (MCL) algorithm (granularity = 4). Bubble charts were plotted by strength of the enrichment effect as the log10 observed/expected (O/E) ratio, where ‘observed’ is the number of queried proteins that are annotated in a given term and ‘expected’ accounts for the number of the proteins expected to be annotated in the same term considering a random network of the same size. The size of the bubbles represents the number of validated targets in each pathway, and color gradient reflects the level of the false-positive discovery rate (FDR) significance (−log10). The bubble charts and Venn diagram image were generated using the SRPlot platform. 

### 2.6. Protein-Protein Interaction Network Analysis

To obtain a system-level perspective of the biological networks underlining a common mechanism for both pathogens, we merged the PPI networks previously identified for EV-miRNAs DE by both *P. bivia* and *T. vaginalis*. To this end, PPI networks were merged using Cytoscape (v3.9.1), visualized with yFiles organic layout, and the top-3 clusters were identified by applying MCL algorithm, (granularity = 4). Topological betweenness centrality analysis of the merged network was conducted to screen for the top-10 hub proteins (CytoHubba v0.1) ranked using the maximal clique centrality (MCC) as a scoring method [62]. The betweenness centrality defines the hub proteins (nodes) as the genes which serve the highest number of times as the shortest paths connecting two nodes in the network. This analysis captures the importance of genes by identifying central proteins, which are highly connected and therefore represent bottlenecks in the flow of communication within the network. The centrality estimative strategy is helpful in the search for potential therapeutic targets [63]. 

## 3. Results

### 3.1. Non-Colonized and Colonized Human Vaginal Epithelial Cells Release Exosomes

EVs isolated from the infection model showed positive staining for tetraspanin-30 (CD63) surface protein (Supplementary Figure S1A), with median size average of 147 nm (Supplementary Figure S1B) and range consistent with characteristics of endocytic origin, i.e., exosomes. The cell viability of biological replicates was high (mean 89.3 ± 1.1% SEM), with no significant variation noted between experimental conditions (*p* = 0.6053) (Supplementary Figure S1C). TEM visualization and nanotracking showed no contamination with apoptotic bodies defined as particles with size 1–5 µm [64]. Colonization by *T. vaginalis* induced the highest number of EVs released in the infection model, when compared to baseline and the other experimental conditions (Figure S1B). 

### 3.2. miRNAs-Containing Extracellular Vesicles from Colonized Human Vaginal Epithelial Cells Identified Pathogenic and Healthy Signatures

**Figure 1:**
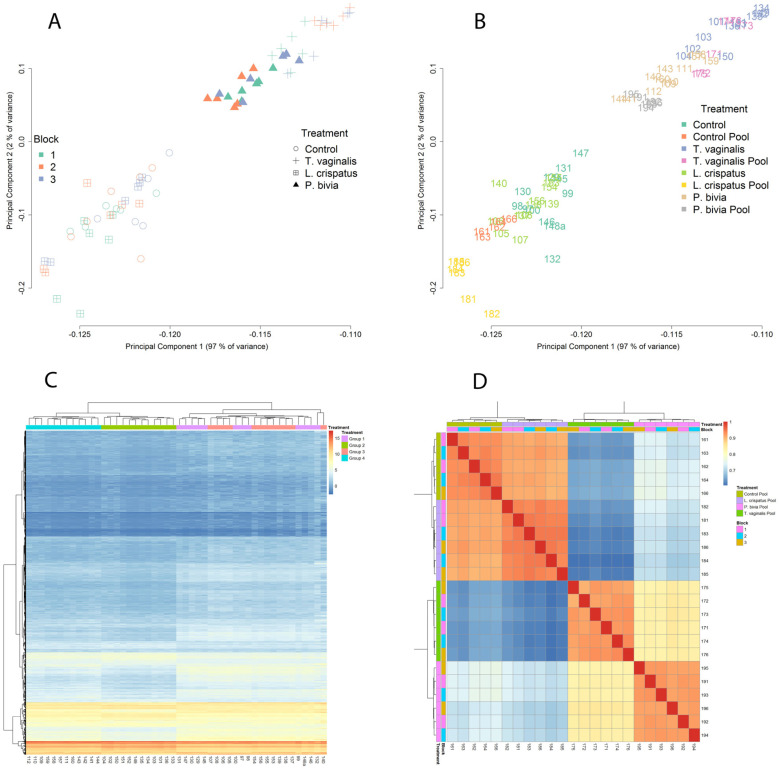
EV-miRNAs expression clustered by type of colonizing organism and differentiated pathogenic from homeostatic condition. Panels A and B illustrate multidimensional scaling. Bivariate plots of biological and technical replicates from 4 independent experiments colored by sequence run (blocks 1–3) (**A**) or by treatment category (**B**) show that the first principal component separates pathogenic (*T. vaginalis* and *P. bivia*) from homeostatic colonization (*L. crispatus*) and baseline control across the X axis and explains 97% of the variance in miRNA expression levels. Panels C and D visualize heatmaps where miRNA expression levels are depicted by variation in color intensity from red (up-regulated) to blue (down-regulated). A hierarchical clustering by treatment (groups 1–4) shows all replicates from control (group 1) and *L. crispatus* (group 3) clustering together and distinctly from *T. vaginalis* and *P. bivia* treatments (groups 2 and 4, respectively) (**C**). A heatmap of the top 500 differentially expressed miRNAs generated from pairwise correlation (Kendall tau) shows high correlation between technical replicates within all sequence runs and well-defined treatment clusters confirming the rigor of differentiating pathogenic from homeostatic conditions by EV-miRNA profiling (**D**).

MSD analysis of the EV-miRNAs transcriptome profiling of all biological and technical replicates revealed two main clusters, which grouped *L. crispatus- colonized* with non-colonized vaginal cells separately from the single *T. vaginalis* and *P. bivia* cluster. These two clusters explained 97% of the variance as a single principal component (Figure 1A,B). This pattern identified similarities among pathogenic colonization and contrasted it with homeostatic bacteria consistent with our leading hypothesis. No obvious batch effect was observed (Figure 1A, runs/blocks 1–3), with pools and individual samples in all runs representing very similar patterns of expression (Figure 1B) and with cluster similarities assembled within the corresponding biological groups. A global heatmap expression profile of the EV-miRNAs resembled the MDS analysis, with 2 major clusters representing a “healthy” versus “non-healthy” (pathogen-triggered) expression profile (Figure 1C). The heatmap of pairwise-correlated (Kendall tau) replicates also demonstrated consistent clustering within the experimental groups with no significant run/block variations (Figure 1D). 

**Figure 2:**
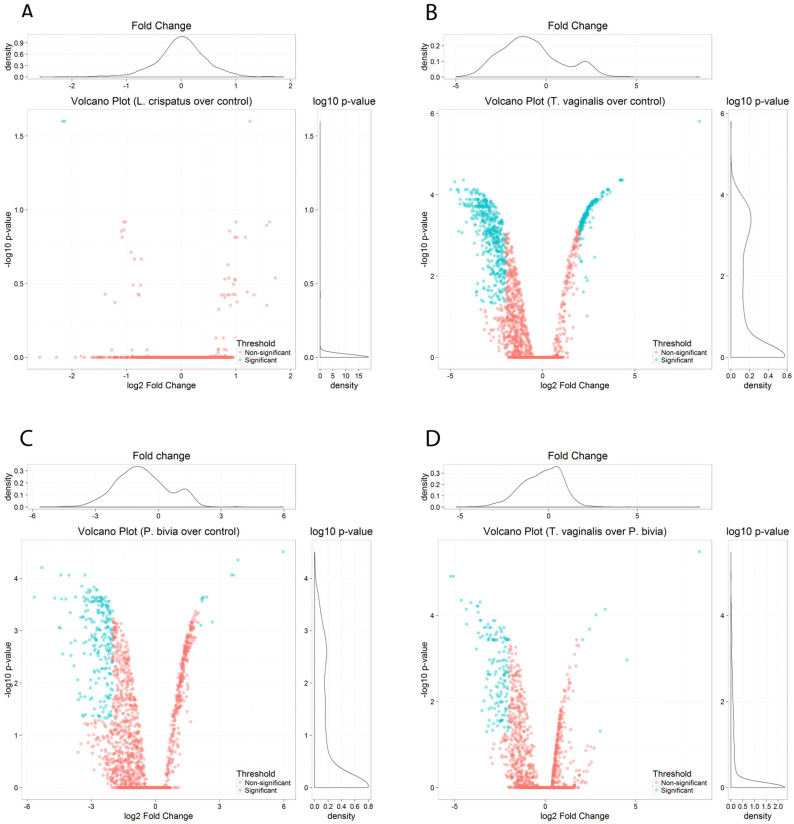
EV-miRNAs were differentially perturbed in response to vaginal challenge with *T. vaginalis* or BV-pathobiont while colonization by *L. crispatus* maintained a homeostatic equilibrium. Volcano plots show miRNAs differentially expressed by pairwise comparison of: (**A**) *L. crispatus* over control, (**B**) *T. vaginalis* over control, (**C**) *P. bivia* over control, and (**D**) *T. vaginalis* over *P. bivia*, depicting the log10 adjusted *p* value versus log2 fold change (FC) for each probe. Significance cut-off was set to adjusted *p* < 0.05 and log FC > 2: blue dots mark EV-miRNAs down- (to the left) and up- (to the right) regulated upon vaginal cell colonization. Pink dots mark non-significant miRNAs.

Volcano plots of pairwise comparisons illustrate differences between the homeostatic *L. crispatus* colonization and pathogenic colonization by *T. vaginalis* and *P. bivia*. No substantial differences were seen between *L. crispatus*-colonized and non-colonized vaginal cells (Figure 2A). *L. crispatus* downregulated only two EV-miRNAs (hsa-miR-3197 and hsa-miR-6845-5p, log2 FC > 2), both with poor annotation, and upregulated one miRNA (hsa-miR-1273e, log2 FC < 2), which has been removed from the most recent miRNA database (miRBase R22.1). In contrast, colonization by *T. vaginalis* and *P. bivia* triggered a striking perturbation in the EV-miRNA cargo compared to non-colonized vaginal epithelial cells (Figure 2B,C) and were distinguished from each other mostly by downregulated miRNAs (Figure 2D). Overall, we identified 938 DE EV-miRNAs induced by *T. vaginalis* and 735 DE *P. bivia* miRNAs with adjusted *p* < 0.05 (Supplementary Tables S1 and S2).

### 3.3. Vaginal Epithelial Cell Colonization by T. vaginalis and the BV-Pathobiont Identified EV-miRNA Targeted Genes and Pathways Associated with Cancer, Viral Infections, and Potential Reproductive Tract Tissue Recipients

**Figure 3:**
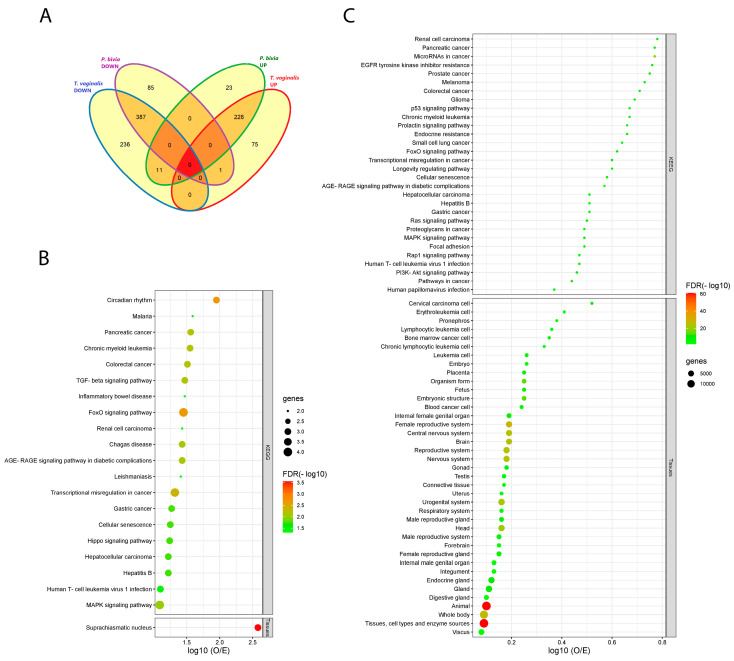
*T. vaginalis* and *P. bivia* triggered similar patterns of EV-miRNAs dysregulation, linking both to common pathway signatures in cancer and reproductive tissues. (**A**) Venn diagram depicts the number of shared and unique EV-miRNAs modulated by *T. vaginalis* and *P. bivia* in each category. (**B**) Total terms enriched in KEGG and in TISSUES respective to shared down-regulated EV-miRNAs targets. (**C**) Top-30 terms enriched in KEGG, and the totality of terms found in TISSUES respective to shared up-regulated EV-miRNAs targets. Bubble charts in B and C portray enriched pathways generated in STRING for the validated targets of EV-miRNAs up- (228) or down-regulated (387) by both *T. vaginalis* and *P. bivia.* Pathways were ranked by strength of the enrichment effect as the log10 (O/E) ratio. The size of the bubbles represents the number of validated targets in each pathway. Color gradient reflects the level of the false discovery rate (FDR) significance (−log10).

Venn diagram analysis of EV-miRNAs differentially expressed (adjusted *p*-value < 0.05) in response to *T. vaginalis* and/or *P. bivia* illustrates four intersecting (shared) and four unique (non-shared) miRNA categories (Figure 3A, Supplementary Table S2). Consistent with our hypothesis for a synergistic relationship between *T. vaginalis* and BV, most DE miRNAs in our model (n = 615) were shared between *T. vaginalis* and *P. bivia* acting in the same direction of dysregulation, with overlapping sets of 228 up-regulated and 387 down-regulated DE miRNAs. On its own, *T. vaginalis* induced the up- or down-regulation of 75 and 236 miRNAs, respectively, while *P. bivia*—23 and 85 miRNAs, respectively. Additionally, 12 discrepantly dysregulated miRNAs were modulated by both pathogens, but in opposite directions. 

Next, we identified validated miRNA target genes and pathways enriched for each of the eight individual (unique) or overlapping (shared) categories described in Figure 3A. miRWalk identified 17 validated target genes for the shared down-regulated miRNA set and 1174—for the shared up-regulated miRNA set (Supplementary Table S3). No validated targets were observed for the 12 discrepantly dysregulated miRNAs. The genes targeted by the shared and uniquely dysregulated miRNAs were used to find enriched pathways in KEGG and to construct PPI networks. Moreover, because EV enable cell-cell communication, we included the database TISSUES in our search strategy to determine potential EV recipients of interest.

Shared down-modulated EV-miRNAs specified a total of 20 significant KEGG pathways (false discovery rate, FDR < 0.05). Five of those have been associated with infectious disease (malaria, Chagas disease, leishmaniasis, hepatitis B and HTLV-1) and five with cancer, corresponding to 50% of the total pathways (Figure 3B). Overall, TGFBR2 accounted for most enriched pathways in KEGG. Additionally, TISSUES identified suprachiasmatic nucleus as potential tissue-target, pinpointing genes, specially CRY2, associated with circadian regulation, a top-1 pathway (FDR < 0.0022) also identified by KEGG (Figure 3B). 

Target genes for shared up-regulated miRNAs revealed 106 KEGG pathways (Supplementary Table S4, Figure 3C) and 39 terms in TISSUES (Figure 3C) (FDR < 0.005). Approximately, 30% of these shared KEGG enriched pathways are related to both cancer and viral infection. TISSUES underlined 13 (33%) terms associated with the female reproductive tract plus pregnancy, and 4 terms (10%) related with the central nervous system.

Non-shared EV-miRNAs dysregulated by *P. bivia* identified 3 downregulated and 83 upregulated target proteins (Supplementary Figure S2A,B and Table S3). The targets for up-regulated miRNAs were enriched in 16 KEGG pathways, highlighting 6 (37.5%) pathways in cancer (Supplementary Figure S2A). Targets for down-regulated miRNAs were not informative (Supplementary Figure S2B). Additionally, *T. vaginalis* unique up-/down-regulated EV-miRNAs identified 148 and 94 targets, respectively (Supplementary Figure S2C,D and Table S3). Targets from the up-regulated miRNAs identified 40 KEGG pathways (FDR < 0.05), including 16 (40%) terms in cancer and 9 (22.5%) terms in viral infection. Furthermore, the 25 terms in TISSUES specified were linked to cancer (8/32%), the female genital tract and fetus (8/32%), and the central nervous system (2/8%) (Supplemental Figure S2C). miRNAs downregulated by *T. vaginalis* were less informative yet underlined the female reproductive system (TISSUES, FDR = 0.00039) (Figure S2D). Altogether, terms and pathways individually found for each pathogen largely reiterate the pattern reported for the shared up/down-regulated networks, implicating cancer, the female reproductive tract, and infectious diseases.

### 3.4. miRNA Dysregulation by Parasitic and BV-Associated Organisms Targets Steroid Hormone Receptor Signaling and Pathways Associated with Cancer, Viral Infections, and Potential Reproductive Tract Tissue Recipients

**Figure 4:**
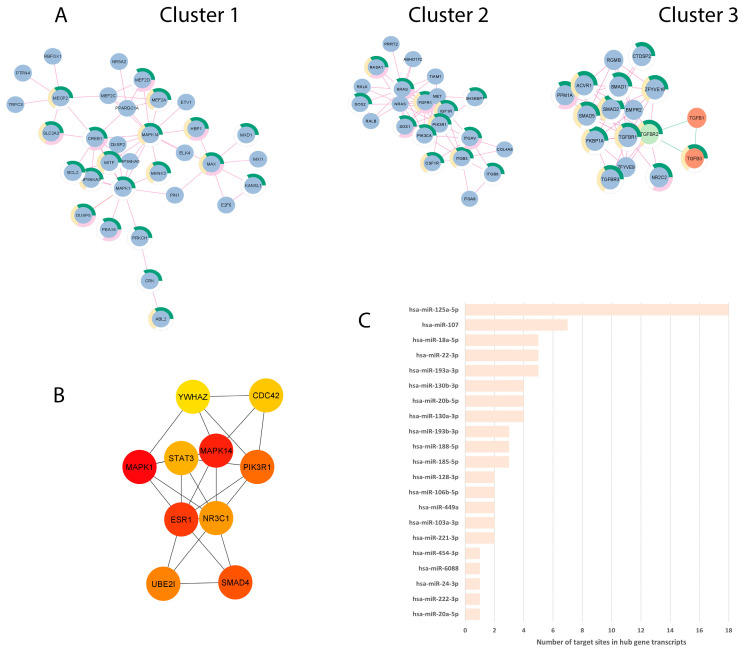
PPI network analysis identified top 3 clusters and 10 hub genes dysregulated by both *T. vaginalis* and *P. bivia*. (**A**) Top three main clusters identified by Cytoscape: Blue and orange circles (nodes) depict putatively down- and up-regulated proteins, respectively. TGFBR2 (green node) had its expression predicted in both directions. Edges represent interactions of proteins estimated from up-regulated (pink lines) or down-regulated (green lines) miRNAs data sets. Cluster 1:33 nodes and 55 edges, cluster 2:22 nodes and 58 edges, and cluster 3:17 nodes and 48 edges (proteins are shown in Supplementary Table S5). Donut semicircles show proteins enriched in terms related to the female reproductive system (green), female reproductive gland (pink), and internal female genital organs (yellow) (TISSUES database). (**B**) PPI between the top-10 hub genes identified from the merged network, determined by CytoHubba according to MCC analysis. Colors depict centrality score from higher (red) to lower (yellow) values (values are shown in Table 1). (**C**) EV-miRNAs targeting the 10 hub genes, plotted according to the total number of 3′UTR target sites including splicing variants (respective expression values are shown in Supplementary Table S7).

To explore the synergism of the networks determined by the EV-miRNAs DE by both *T. vaginalis* and *P. bivia*, we merged the two validated-targets constructed PPI networks obtained from up- and down-regulated EV-miRNAs and screened for top 10 hub genes. The merged network comprehended 1184 nodes and 1710 edges (*p* = 1.0^−16^), of which the top-3 clusters contained a total of 72 proteins, 46 of them associated with terms on female genital tract (Figure 4A, Supplementary Table S5). The 10 hub genes identified from the global merged network are protein targets determined by the shared up-regulated miRNAs set, and we found them to be associated with signal transduction (MPK1, MPK14, PIK3R1, SMAD4, YWHAZ), gene transcription (STAT3), cell division (CDC42), ubiquitination (UBE2I), and steroid hormone receptors/transcription factors (ESR1, NR3C1) (Figure 4B, Table 1). The PPI between the hub genes is depicted according to the centrality score (Figure 4B), and respective miRNA-targeted hub genes (Figure 4C, Table 1). We found 21 EV-miRNAs modulating the hub genes, varying by the number of miRNAs per mRNA-target (Table 1) and the number of 3′UTR target sites in any of the transcripts, including splicing variants (Figure 4C and Table 1). The top 10 hub genes were ranked in importance for the merged network by centrality score (representing essential proteins in the network, which can be therapeutic targets) each showing a high degree of PPI interaction (number of links to given node ranging from 53 to 24) (Table 1) and each occurring in multiple terms related with the urogenital and female reproductive tract in TISSUES (Supplementary Table S6).

The hub gene, which was targeted by the highest number of *T. vaginalis*- and BV pathobiont-upregulated EV-miRNAs and ranked third in importance for the merged network, was the estrogen receptor 1 (ESR1, Table 1), which is central for the reproductive female physiology [65]. The top ranking also identified the potential downregulation of other genes involved in the ESR1 pathway (Supplemental Figure S3), including signal transduction MAPK1/ERK2 and the enzyme PIK3R1 (and Table 1). In addition, among the shared upregulated EV-miRNAs was hsa-miR-18a-5p, which targets both ESR1 and isoforms of the glucocorticoid receptor (GR) gene NR3C1 (GRα and GRγ), acting to decrease their levels. NR3C1 was ranked seventh by centrality score in the pathogen-dysregulated PPI network (Table 1).

## 4. Discussion

**Figure 5:**
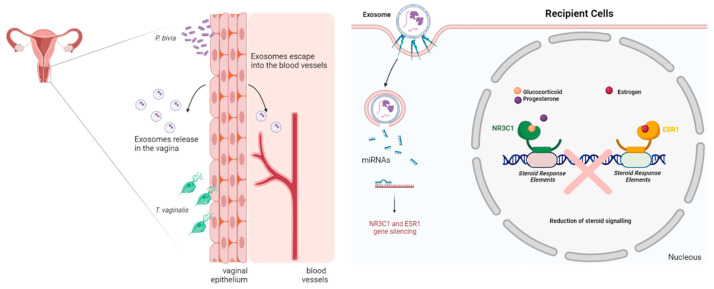
Summary of conceptual framework for testing the causative link between parasite/bacterial colonization and post-transcriptional regulation of human female reproductive function and homeostasis in reproductive tissues. The model relies on the ability of human vaginal epithelial cells to mount differential miRNA expression in response to pathogenic or homeostatic colonization by a common parasite (*T. vaginalis*), a signature BV pathobiont (*P. bivia*) or signature vaginal health bacteria (*L. crispatus*). Differentially expressed miRNAs will be released encapsuled in extracellular vesicles (EV or exosomes) to potentially exert paracrine or systemic/endocrine function in recipient tissues. Quantifying EV-miRNAs with predicted reproductive tissues and validated gene targets and pathways, such as steroid hormone signaling or gynecologic cancer, supports molecular causation (Image created with BioRender.com, accessed on 19 December 2022).

The experimental data presented here support our hypothesis conceptualized in Figure 5.

To our knowledge, this study is the first to investigate the effect of *T. vaginalis* infection on host miRNAs released in EVs in the context of vaginal infections as potential pathogenic determinant. *T. vaginalis* is an extracellular protozoan parasite that adheres to the vaginal and cervical epithelial cells and subverts host immune defenses by signaling through its surface lipophosphoglycan and release of endosymbiont virus (TVV) and exosomes [32,33] loaded with specific protein cargo, which can be modified by TVV [35]. For our first proof of concept study, we chose a strain that harbors TVV to represent most primary *T. vaginalis* clinical isolates worldwide [66]. 

Our study is also the first to investigate EV-miRNAs differential expression in response to axenic *P. bivia* vaginal colonization and to contrast that to *L. crispatus* colonization. *P. bivia* is an abundant pathobiont of the vaginal microbiome community state CST-IV, subtype A [67], which presents BV-related characteristics, and a close to 2-fold increased risk of acquiring *T. vaginalis* infection [18]. Both pathogens, *T. vaginalis* and *P. bivia*, uphold clinical relevance associated with poor perinatal outcomes, urogenital cancer, and increased susceptibility to viral STIs [6]. We found that all miRNAs dysregulated by *T. vaginalis* and or *P. bivia*, remained at baseline equilibrium after colonization by *L. crispatus*. In fact, the lactobacillus challenge was associated with DE of only two miRNAs with poor annotation and no assigned function by the latest mRNA databases. These findings provide further molecular proof for the previously proposed beneficial role of *L. crispatus* in vaginal health [6,10]. While EVs produced by vaginal *Lactobacillus* isolates have been reported to have a protective effect against HIV-1 transmission, in part, by inhibiting viral attachment/entry due to diminished exposure of viral envelope [68], the individual components of the EV cargo responsible for the causation of the reported protective effects are still to be determined. 

Our experimental study provides a causative proof for clinical findings of disturbed vaginal miRNome. In our model, host EV-miRNAs expression profile of 2078 miRNA probes strikingly distinguished homeostatic from pathobiont human vaginal epithelial cells colonization, both PCA and heat-map analyses clustering together on one side *T. vaginalis* and *P. bivia* and on the other side—*L. crispatus* and baseline vaginal cells. A Swedish study of 56 reproductive age women surveying only 798 miRNAs identified 10 miRNAs overexpressed in non-Lactobacillus dominated microbiome communities with specificity over 95% in receiver operating characteristics (ROC) analysis [69]. All of those miRNAs proposed to have a high diagnostic potential (except miR-4532 which was later determined to not be a true miRNA) including miR-23a-3p, miR-130a-3p, miR-22-3p, miR-1290, miR-15a-5p, miR-1537-3p, miR-222-3p, miR-27a-3p, and miR-148a-3p, were upregulated by the pathogens in our model (7 upregulated by both *T. vaginalis* and *P. bivia*, one by *T. vaginalis* alone and one by *P. bivia* alone, Supplementary Table S2). The top 2 dysbiotic vaginal miRNAs with a predictive value validated in another cohort of 32 women (miR-23a-3p and miR-130a-3p), were present in our dataset among the dysregulated miRNAs targeting four different 3′UTR sites of the ESR1, which was identified as one of the top 10 hub genes in the PPI network analysis discussed below. These clinical findings support our conceptual framework and the relevance of our experimental model linking vaginal microbes to miRNA dysregulation. 

Our *in silico* analysis of miRNA-predicted pathways and PPIs applied a stringent and conservative approach focusing on the 3′UTR targeting sites validated and confirmed by three algorithms. The miRNA pairing with 3′UTR sites follows the canonical pathway of miRNA regulation where overexpressed miRNAs downregulate their targets and down-expressed miRNAs allow their targets expression, without assumptions about ‘on/off’ switch, tuning, or neutral effects on protein levels [70]. 

It is noteworthy that *T. vaginalis* and *P. bivia* caused a massive, shared repression of 387 EV-miRNAs; however, miRWalk *in silico* analysis of miRNA targets was more informative for the set of 236 up-regulated EV-miRNAs, suggesting unknown roles for most EV-miRNAs identified as downregulated. EV-miRNAs targets identified by miRWalk were used to build up PPI networks and to find, by GSEA, a germane group-based gene set with a higher likelihood of identifying either attractive therapeutic targets or biological processes relevant to our hypothesis. In addition, to obtain a global perspective of EV-miRNAs targets, we merged PPI networks from shared up- and down-regulated EV-miRNAs and identified top-3 clusters of the PPI, which showed 46 out of 72 proteins (~63.8%) related with FGT terms in TISSUES. Furthermore, utilizing profiles of EV-miRNAs modulated by both pathogens, GSEA showed distinctive proteins and pathways mainly associated with cancer. Our findings were consistent with the so far sparse evidence of clinical relevance of miRNAs to FGT cancer. A clinical study of cervical cancer reported hsa-miR-21 and hsa-miR-146a to be overrepresented in exosomes from cervicovaginal lavages [71], and miR-92a-5p and miR-155-5p were pointed out as biomarkers for early detection of cervical cancer using pap smear samples—considering the nature of the samples, this data may reflect the collective of miRNAs from cells plus exosomes [72]. In our data, hsa-miR-21-5p and hsa-miR-92a-3p (the hsa-miR-92a-5p passenger strand) were up-regulated by both *T. vaginalis* and *P. bivia* and hsa-miR-146a-5p was up regulated by *P. bivia.* While *in silico* analysis fails to correlate hsa-miR-21-5p with proteins assigned to cancer pathways, it links hsa-miR-146a-5p to cancer by targeting ERBB4, a protein enriched in two KEGG terms associated with cancer (‘ErbB signaling pathway’ and ‘proteoglycans in cancer’). Viral infection-associated cancer pathways were predicted by shared up-regulated networks as well as EV-miRNAs up-regulated by *T. vaginalis* only. Thus, our experimental findings and predictions agree with clinically identified miRNAs and observational studies associating BV and trichomoniasis with viral cancer [14,15,20,73,74].

A merged PPI network was used to find the top-10 hub genes and their respective targeting EV-miRNAs because network nodes with higher betweenness centralities (hubs), connecting different distinct functional modules, are more likely to be essential than high-degree nodes [75] and have a higher potential to be therapeutic targets [63]. By employing this approach, we found 10 hub genes involved with cell signaling (MPK1, MPK14, PIK3R1, SMAD4, YWHAZ), cell division (CDC42), and ubiquitination (UBE2I), along with gene transcriptional factors (STAT3, ESR1, and NR3C1) where two of them are also hormone steroid receptors, ESR1(ERα) and NR3C1 (GR). The steroid receptors might be of special relevance to our hypothesis due to proposed roles of estrogens in collaborating with lactobacilli-dominated microbiome [76], and ESR1 and NR3C1 as genetic factors of PTB [77], and GR epigenetic modification linked to poor perinatal clinical outcomes [78,79]. 

Among the hub genes, ESR1 is the transcript (variants 1–4 and 7) targeted by the highest number of miRNAs and presents the highest number of targeted 3′UTR sites. The redundancy in targeting ESR1 may reflect the importance for both pathogens in controlling this receptor in EVs recipient cells and, thus, manipulating the conspicuous role of estrogens in the FGT, a conjecture that warrants further investigation. ESR1 is mainly expressed in uterus, ovaries, breast, kidney, liver, white adipose tissue, and bones. It is highly homologous to ESR2 (ERβ) which, in turn, is found in male and female reproductive organs, central nervous system (CNS), cardiovascular system, lung, immune system, colon, and kidney [80]. In addition, ESR1 plays an important role in many immune cells and cancer [81], a connection that needs further elucidation in the context of *T. vaginalis* and BV-associated cancer in reproductive systems. Estrogens have known anti-inflammatory properties [82]. Therefore, the decreased levels of ESR1 due to 3′UTR pairing with miRNAs differentially upregulated by *T. vaginalis* and BV bacteria may contribute to exacerbating inflammation-driven comorbidities, cancer, adverse pregnancy outcomes. It may also shift balance to proinflammatory activation in other low-estrogen conditions such as menopause. 

Additional attention deserves NR3C1, which has been linked to spontaneous preterm birth [77] and was identified in our dataset as a hub gene with the isoforms GRα and GRγ targeted by hsa-miR-18a-5p, an EV-miRNA upregulated by both *T. vaginalis* and *P. bivia*. Glucocorticoid receptors comprehend a large cohort of protein isoforms that arise from alternative splicing of one single gene (*NR3C1*) and alternative translational initiation mechanisms. GR may act to repress or induce gene expression depending on the receptor isoform, cell type and promoter context, which will ultimately dictate distinct responses to glucocorticoids [83]. Both isoforms are largely expressed and functional; however, they regulate a specific set of glucocorticoid-responsive genes [84], suggesting their downregulation may result in different outcomes based on the isoform itself and EV-recipient cells. NR3C1 methylation status has been pointed to as a factor for adverse experiences in perinatal period, where hypermethylation of certain CpG sites correlates with perinatal stress occurrence [79,85]. NR3C1 methylation leads to attenuation of GR expression, impairing the negative feedback loop of the hypothalamic-pituitary-adrenal (HPA) axis, resulting in an increase in glucocorticoid levels—such as cortisol—which in turn may affect the fetus’ brain development [86]. Interestingly, NR3C1 seems to be convergent to insulin growth factor 2 (IGF2) in the context of cell responses to stress [86]. IGF2 signals through insulin-like growth factor 1 receptor (IGFR1), activating PI3K/MAPK intracellular signaling. IGF and its receptors are involved in fetus and proper placenta development [87]. Here we found IGFR1 and PI3K as part of cluster 2 and MAPK proteins as part of cluster 1, hence, we hypothesize that downregulation of NR3C1 and IGFR1 axis, and respective signaling molecules, may help to explain long-lasting adverse prenatal events associated with *P. bivia* and *T. vaginalis* infection, pointing to a possible post-transcriptional regulation by EV-miRNAs in addition to CpG methylation. EVs act in recipient cells, possibly targeting bystander cells or traveling to reach long-distance cells; thus, it is plausible that EV-miRNAs dysregulated by vaginal pathogens could target surrounding tissues, such as the cervix, and distant ones such as the uterus, placenta, and fetus. In our dataset we found enrichment of validated targets in tissues related to the FRT, pregnancy (embryo, fetus, placenta, and embryonic structures), and brain (‘CNS’, ‘brain’ and ‘nervous system’), which support our hypothesis and assign candidate proteins to enlighten possible pregnancy poor outcomes linked with BV. It is worth mentioning that progesterone, a low affinity agonist to GR, maintains pregnancy and favors maternal immune tolerance to fetus via GR dependent mechanism [88], raising the question of how GR downregulation may impact some of the progesterone activity during pregnancy. 

The relevance of the remaining hub genes identified by our analysis of dysbiosis and *T. vaginalis* dysregulated miRNAs (SMAD4, UBE2I, STAT3, CDC41 and YWHAZ) remains to be investigated in the clinical context of *T. vaginalis*, BV and associated cancer and pregnancy complications. Of note, among the top 10 hub genes, STAT3 was the transcript with the highest number of predicted 3′UTR target sites, being a target of hsa-miR-125a-5p. STAT3 is transcriptionally hyperactive in numerous tumor cells and serves as an immune checkpoint in immune-associated tumor cells [89,90]. Serum exosomal studies from cervical cancer and healthy women pinpointed this miRNA as a potential biomarker with diagnostic value [91].

Circadian rhythm was the top-1 KEGG term respective to shared down-regulated EV-miRNAs network. Interestingly, ESR1 and ESR2 have been found to regulate circadian rhythm in mice [92]. Although our *in silico* analysis did not implicate ESR1 in circadian pathway, we found other clock genes targeted by miRNAs dysregulated by *T. vaginalis* and *P. bivia* including PER2, CLOCK, and CRY2. The literature is limited on ESR and circadian rhythm; so far it seems there is a link between ESR1-induced CLOCK expression in breast cancer cells [93]. Furthermore, the same network identified KEGG terms related with protozoan infection, such as malaria, Chagas disease and leishmaniosis, pointing to a convergent set of genes (TGFB1, TGFB3, and TGFBR2) related with those infections and BV. It has been appreciated that sex-associated hormones have a role in the susceptibility to some protozoan infections [94]. Yet, ESR1 and NR3C1 were not associated with terms in the protozoan dataset; instead, we found TGFB1 and TGFB3—expected to be up-regulated—associated with the three protozoans mentioned above, and TGFBR2 only linked with Chagas diseases. TGFBR2 is a predicted target for both up- and down-regulated set of EV-miRNAs, requiring more investigations regarding gene expression regulation. 

## 5. Conclusions

Our data support the main hypothesis and emerging concept that parasitic eukaryotes and bacterial pathogens disturbing the human vaginal microbiome may impact reproductive health by modifying the EV-miRNAs cargo. Our data provide a causative proof for a homeostatic role of *L. crispatus* and in contrast—a host transcriptome disruptive role of the common vaginal parasite *T. vaginalis* and the BV-signature pathobiont *P. bivia*. In silico analysis of the experimental EV-miRNA transcriptome patterns predicted perturbing effects of pathogenic colonization in networks and pathways closely related with steroid hormone receptors functioning in the female reproductive physiology. While emerging clinical science supports the role of vaginal bacteria in altering the miRNA cargo released in the cervicovaginal secretions [69] and suggests that miRNAs dysregulation, which we experimentally linked to *P. bivia* and *T. vaginalis,* may contribute to risks of gynecologic cancers associated with these pathogens, more research is needed to validate their miRNA-mediated impact on protein networks within cancer cells and tissues. Future studies should also focus on clinical validation of experimentally derived miRNA signatures under conditions of BV and mixed infections and predicted effects on steroid hormone signaling while controlling for covariates that cannot be reproduced *ex vivo*. In addition, a development of new tools/algorithms enabling the investigation of human EV-miRNAs targeting parasite genes would advance our knowledge of host-parasite interactions.

## Figures and Tables

**Table 1:** Top-10 hub genes identified in the merged PPI network and correspondent targeting EV-miRNAs.

Hub Rank	Gene Symbol	Gene Name	Centrality Score *	Degree **	EV-miRNAs Targeting Hub Genes	Number of 3′UTR Target Sites
1	MAPK1	mitogen-activated protein kinase 1	26,313.7	53	hsa-miR-106b-5p, hsa-miR-20a-5p, hsa-miR-6088	4
2	MAPK14	mitogen-activated protein kinase 14	25,464.1	40	hsa-miR-24-3p	1
3	ESR1	estrogen receptor 1	24,999	34	hsa-miR-130a-3p, hsa-miR-130b-3p, hsa-miR-18a-5p, hsa-miR-20b-5p, hsa-miR-22-3p, hsa-miR-221-3p, hsa-miR-222-3p, hsa-miR-454-3p	22
4	SMAD4	SMAD family member 4	22,813.3	41	hsa-miR-449a	2
5	PIK3R1	phosphoinositide-3-kinase regulatory subunit 1	19,971.2	46	hsa-miR-103a-3p, hsa-miR-107, hsa-miR-128-3p	11
6	UBE2I	Ubiquitin Conjugating Enzyme E2 I	19,821.3	32	hsa-miR-188-5p	3
7	NR3C1	nuclear receptor subfamily 3 group C member 1	19,570.7	24	hsa-miR-18a-5p	4
8	STAT3	signal transducer and activator of transcription 3	17,914.2	39	hsa-miR-125a-5p	18
9	CDC42	cell division cycle 42	16,943.4	34	hsa-miR-185-5p	3
10	YWHAZ	tyrosine 3-monooxygenase/tryptophan 5-monooxygenase activation protein zeta	15,952.3	29	hsa-miR-193a-3p, hsa-miR-193b-3p	8

* Centrality score reflects the number of times a particular node is the shortest path between another two nodes. High score values represent essential proteins in the network, which can be potential therapeutic targets. ** Degree is the number of links to a given node. PPI—protein-protein interaction.

## Data Availability

The data discussed in this publication have been deposited in NCBI’s Gene Expression Omnibus [95] and are accessible through GEO Series accession number GSExxx (https://www.ncbi.nlm.nih.gov/geo/query/acc.cgi?acc=GSExxx accessed on 19 December 2022).

## Supplementary Materials

The following supporting information can be downloaded at: https://www.mdpi.com/article/10.3390/microorganisms11030551/s1, Figure S1. Isolated particles met the size criteria for extracellular vesicles of endocytic origin; Figure S2. Enriched pathways signatures of EV-miRNAs modulated by T. vaginalis or P. bivia; Figure S3: EV-miRNAs target proteins in the estrogen signaling pathway; Table S1: DE EV-miRNAs; Table S2: Venn-diagram miRNA; Table S3: miRNA targets Pb-Tv; Table S4: KEGG tissues shared Tv-Pb UP_miRNA; Table S5: genes top-3 clusters; Table S6: Occurrence of the 10 hub genes in terms related with female reproductive tract annotated in TISSUES database; Table S7: Expression values of EV-miRNAs targeting hub genes.
