# Supplementary material for: The microRNA Cargo of Human Vaginal Extracellular Vesicles Differentiates Parasitic and Pathobiont Infections from Colonization by Homeostatic Bacteria
Source: Microorganisms. 2023 Feb 21;11(3):551. doi: 10.3390/microorganisms11030551 (PMC10054151; doi:10.3390/microorganisms11030551)
Supplement: Supplementary file 1 [file microorganisms-11-00551-s001.zip › supplemental_info_final-20230221/Suppplemental-FiguresS1-4&TablesS6-7.pdf]

SUPPLEMENTARY MATERIALS

FIGURES

A

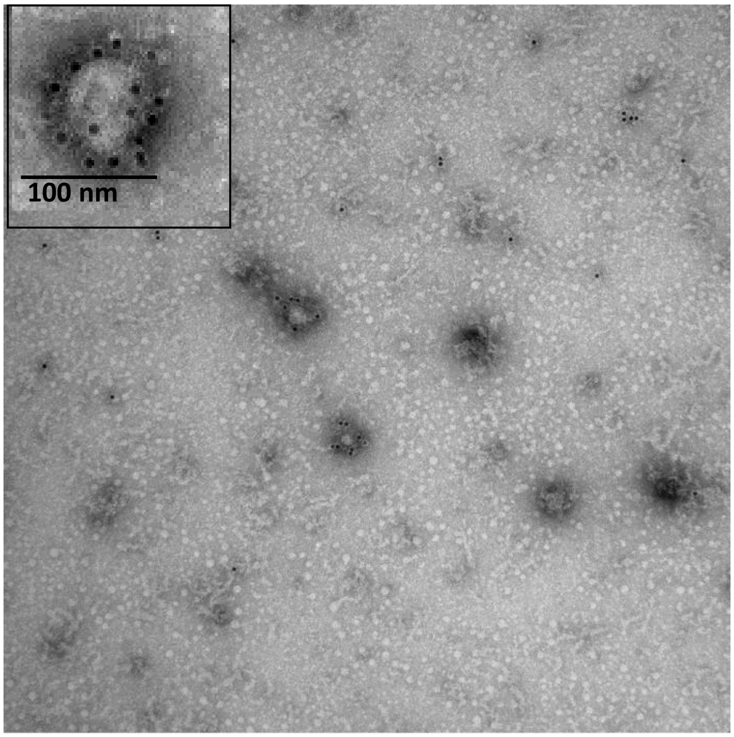

B

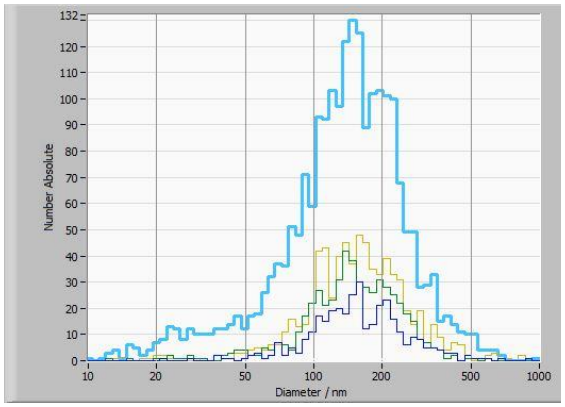

C

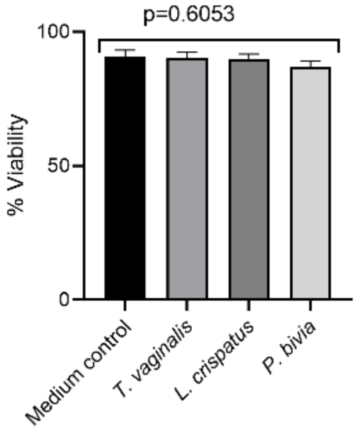

Figure S1. Isolated particles met the size criteria for extracellular vesicles (EV) of endocytic origin. (A) Wide-field and close-up (insert upper left corner) representative electron-microscopy images of CD63+ (tetraspanin-30) vaginal EVs. After isolation from colonized and control vaginal epithelial cultures, EVs were fixed and stained with anti-human CD63 antibody, followed by staining with secondary bridging antibody. Then, probed with protein A-gold (5nm). The grids were examined in a TecnaiG2 Spirit BioTWIN microscope and images were acquired with an AMT 2k CCD camera. (B) Nanoparticle tracking analysis was performed with ZetaView, using samples under the same dilution factor (50X). Image shows the number absolute distribution of small particles per diameter (nm). Distributions present only one peak with EV size range consistent with exosome definition. The experimental groups, respective EV size (median in the weighted distribution) plus the number of traced particles are as follow: light blue = *T. vaginalis* (140 nm / 2525), yellow = *P. bivia* (152 nm / 727), green = *L. crispatus* (145 nm / 579), and dark blue = medium control (151 nm / 569). (C) Cell viability was estimated by Trypan blue exclusion. Bars show means and SEM for a minimum of 6 biological replicates for the conditions listed on the x axis. P value was derived from one-way ordinary ANOVA comparing all conditions.

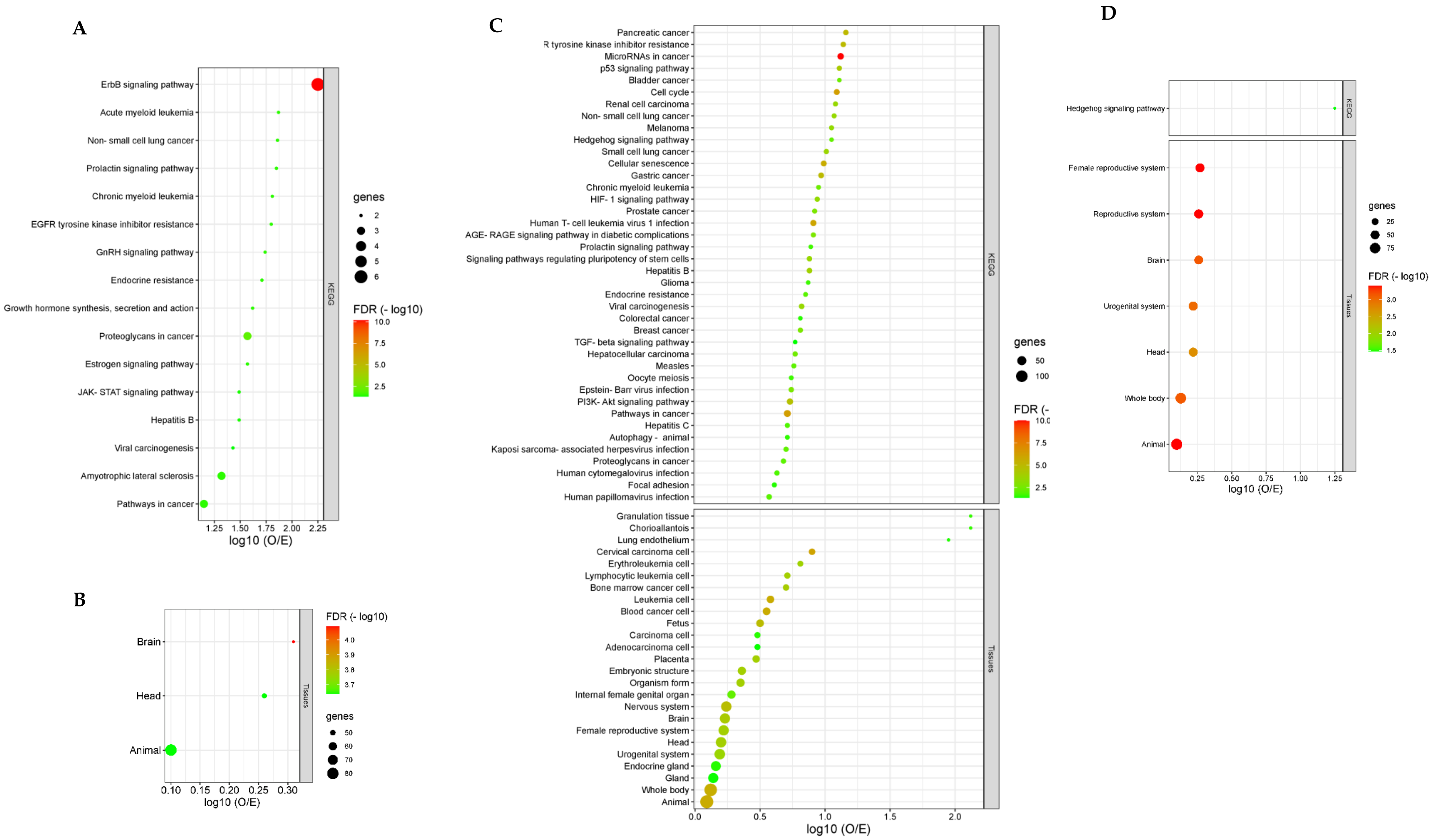

Figure S2. Enriched pathway signatures of EV-miRNAs modulated by *T. vaginalis* or *P. bivia*. EV-miRNAs Up- and down-regulated by *P. bivia* (A – B) or *T. vaginalis* (C – D) were queried in miRWalk to obtain validated targets. The targets were used to construct PPI networks and

perform enrichment pathway analysis using STRING. Here, we shown a complete list of terms found in KEGG and/or TISSUES, in respective to (A) miRNAs up-regulated by *P. bivia* and (B) miRNAs down-regulated by *P. bivia*, (C) miRNAs up-regulated by *T. vaginalis*, (D) miRNAs down-regulated by *T. vaginalis*. Bubble charts portray enriched pathways ranked by strength of the enrichment effect as the log 10 observed/expected (O/E) ratio, where 'observed' is the number of queried proteins that are annotated in a given term and 'expected' accounts for the number of the proteins expected to be annotated in the same term considering a random network of the same size. The size of the bubbles represents the number of validated targets in each pathway. Color gradient reflects the level of the FDR significance (-log10).

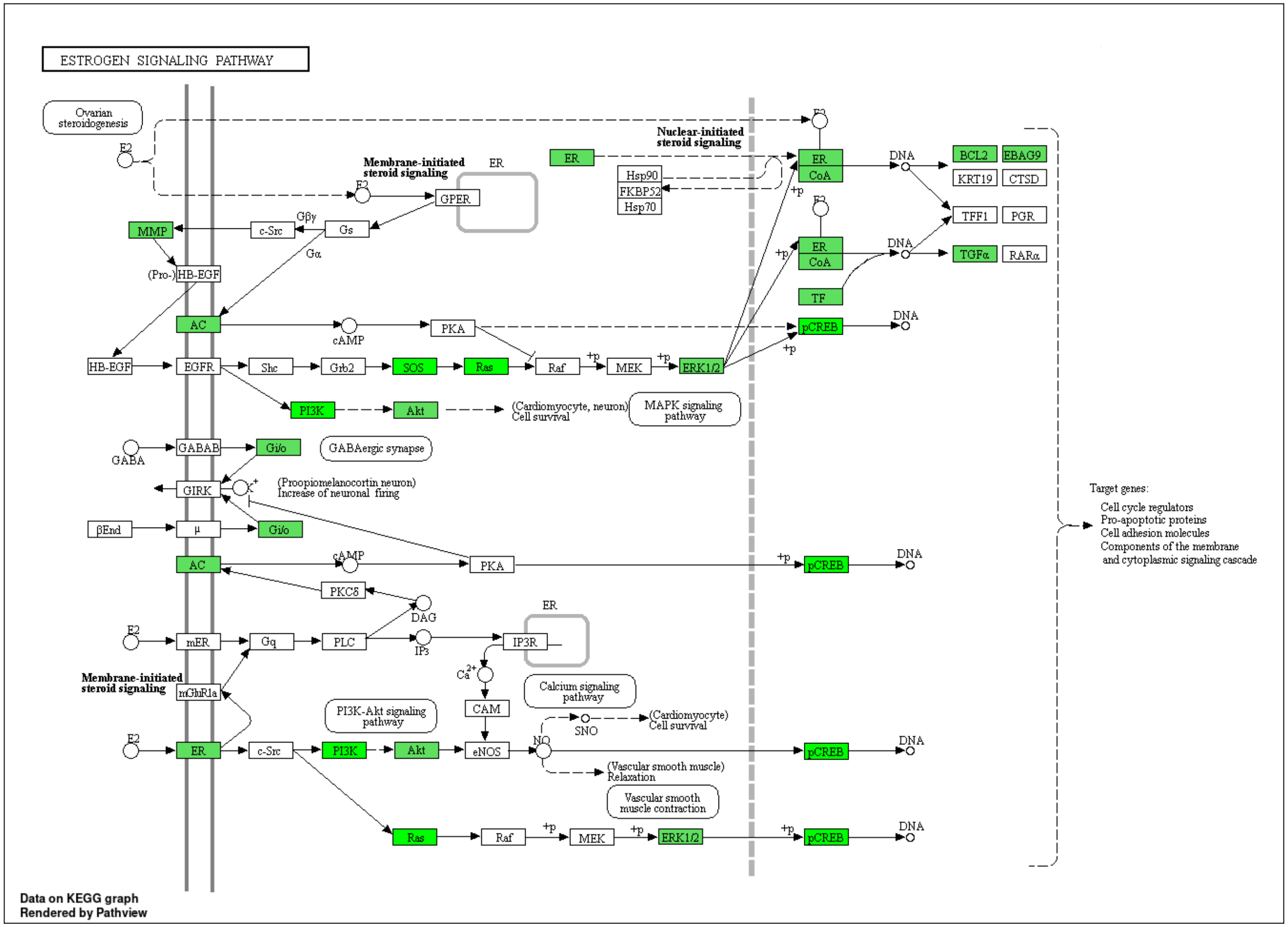

Figure S3: EV-miRNAs target proteins in the estrogen signaling pathway. Target genes of EV-miRNAs up- and down-regulated by both *T. vaginalis* and *P. bivia* were queried to find KEGG pathways in the estrogen signaling pathway. The identified proteins (green) came only from the up-regulated EV-miRNAs gene set.

TABLES

Table S6 – Occurrence of the 10 hub genes in terms related with female reproductive tract annotated in TISSUES database

**hub genes symbol**	**terms in TISSUES / ID**
MAPK1, MAPK14, SMAD4, UBE2I, PIK3R1, STAT3, CDC42, NR3C1, ESR1, YWHAZ	Female reproductive system BTO:0000083
	Reproductive system BTO:0000081
	Urogenital system BTO:0003091
	Internal female genital organ BTO:0003099
STAT3, ESR1, YWHAZ	Female reproductive gland BTO:0000254
CDC42, NR3C1, ESR1, YWHAZ	Uterus BTO:0001424

Table S7 – Expression values of EV-miRNAs targeting hub genes

	T. vaginalis over control		*P. bivia* over control	
**EV-miRNAs**	**log2 FC**	**adjusted p-value**	**log2 FC**	**adjusted p-value**
hsa-miR-103a-3p	2.76	1.50E-04	1.64	9.75E-04
hsa-miR-106b-5p	2.43	2.36E-04	1.40	2.12E-03
hsa-miR-107	2.51	1.94E-04	1.52	1.30E-03
hsa-miR-125a-5p	2.17	4.25E-04	1.02	1.76E-02
hsa-miR-128-3p	2.13	4.75E-04	1.27	4.22E-03
hsa-miR-130a-3p	2.08	7.00E-04	1.20	8.94E-03
hsa-miR-130b-3p	1.98	1.78E-03	1.18	2.15E-02
hsa-miR-185-5p	2.21	3.86E-04	1.31	3.63E-03
hsa-miR-188-5p	2.01	5.82E-04	1.08	1.15E-02
hsa-miR-18a-5p	2.45	2.80E-04	1.43	2.88E-03
hsa-miR-193a-3p	2.18	4.12E-04	1.28	3.33E-03
hsa-miR-193b-3p	2.09	5.21E-04	1.29	3.57E-03
hsa-miR-20a-5p	2.39	2.95E-04	1.29	4.65E-03
hsa-miR-20b-5p	2.30	3.39E-04	1.20	6.73E-03
hsa-miR-221-3p	2.09	5.74E-04	1.26	4.86E-03
hsa-miR-222-3p	2.27	3.90E-04	1.28	5.29E-03
hsa-miR-22-3p	1.98	9.30E-04	1.22	8.46E-03
hsa-miR-24-3p	2.13	4.49E-04	1.36	2.03E-03
hsa-miR-449a	1.81	2.78E-03	1.22	2.75E-02
hsa-miR-454-3p	3.30	8.57E-05	1.88	4.32E-04
hsa-miR-6088	1.90	2.11E-03	1.04	3.05E-02
